# HARE-Mediated Endocytosis of Hyaluronan and Heparin Is Targeted by Different Subsets of Three Endocytic Motifs

**DOI:** 10.1155/2015/524707

**Published:** 2015-03-25

**Authors:** Madhu S. Pandey, Edward N. Harris, Paul H. Weigel

**Affiliations:** ^1^Department of Biochemistry & Molecular Biology, Penn State Hershey College of Medicine, Hershey, PA 17033, USA; ^2^Department of Biochemistry, University of Nebraska, Lincoln, NE 68588, USA; ^3^Department of Biochemistry & Molecular Biology, Oklahoma Center for Medical Glycobiology and University of Oklahoma Health Sciences Center, Oklahoma City, OK 73104, USA

## Abstract

The hyaluronan (HA) receptor for endocytosis (HARE) is a multifunctional recycling clearance receptor for 14 different ligands, including HA and heparin (Hep), which bind to discrete nonoverlapping sites. Four different functional endocytic motifs (*M*) in the cytoplasmic domain (CD) target coated pit mediated uptake: (YSYFRI^2485^ (*M1*), FQHF^2495^ (*M2*), NPLY^2519^ (*M3*), and DPF^2534^ (*M4*)). We previously found (Pandey et al. J. Biol. Chem. 283, 21453, 2008) that *M1*, *M2*, and *M3* mediate endocytosis of HA. Here we assessed the ability of HARE variants with a single-motif deletion or containing only a single motif to endocytose HA or Hep. Single-motif deletion variants lacking *M1, M3*, or *M4* (a different subset than involved in HA uptake) showed decreased Hep endocytosis, although *M3* was the most active; the remaining redundant motifs did not compensate for loss of other motifs. Surprisingly, a HARE CD variant with only *M3* internalized both HA and Hep, whereas variants with either *M2* or *M4* alone did not endocytose either ligand. Internalization of HA and Hep by HARE CD mutants was dynamin-dependent and was inhibited by hyperosmolarity, confirming clathrin-mediated endocytosis. The results indicate a complicated relationship among multiple CD motifs that target coated pit uptake and a more fundamental role for motif *M3*.

## 1. Introduction

Stabilin-2 (Stab2) and HARE (half-length Stab2) function as primary scavenger receptors for the systemic clearance from lymph and blood of hyaluronan (HA) [1], heparin (Hep), and 12 other functionally and structurally distinct ligands [2–6]. HA turnover and catabolism by HARE have been studied extensively for decades [1, 7, 8] and the responsible receptor was molecularly identified >15 years ago after it was purified and cloned [5, 6, 9, 10]. HARE endocytosis of HA occurs over a broad range of sizes from ~2.5 kDa to >MDa [11]. Hep is cleared from the body by two different mechanisms: larger Hep is rapidly cleared from blood in a high-affinity saturable binding mechanism by HARE/Stab2 in liver sinusoidal endothelial cells [12, 13], whereas low mass Hep is primarily cleared by kidney [14] in a nonsaturable renal excretion mechanism [15].

HA and Hep have distinct binding sites within the HARE ectodomain and neither ligand competes for the binding and endocytosis of the other [2]. Several articles in this special issue summarize the many functions of HA. As with HA, the biological and clinical activities of Hep have been studied for decades, and Hep is the most highly prescribed drug in the USA (e.g., for preventing or treating thromboembolic diseases and postsurgery clotting) [16]. HA is synthesized by many cell types and is the longest (up to 5 × 10^4^ sugars) and only unsulfated glycosaminoglycan. In contrast, Hep is synthesized by mast cells as a serglycin proteoglycan with much shorter polysaccharide chains, <50 sugars [17, 18]. Hep is the most anionic glycosaminoglycan, due to extensively sulfated disaccharide isomers and binds to many different matrix, soluble, and cell surface proteins, including growth factors [19]. Hep also functions as a coreceptor or anticoagulant agent [20, 21].

Many receptors require bound ligand in order to interact with adapter proteins and then be targeted to coated pits [22]. In contrast, HARE and other constitutively recycling clearance receptors (e.g., asialoglycoprotein and LDL receptors) are continuously targeted to coated pits, internalized, and recycled back to the cell surface whether bound to ligand or not. Endocytic receptors often contain a tyrosine-based motif (e.g., YXX*φ* or NPXY, where X is any amino acid and *φ* is a hydrophobic residue) or a dileucine motif (D/EXXXLL/I) involved in clathrin-mediated endocytosis [23–26]. YXX*φ* and dileucine motifs interact with AP-2 adaptor complexes, whereas NPXY motifs interact with AP-2 and other adaptor proteins such as Dab2 and ARH [25]. The adaptor protein GULP is required for Stab2-mediated phagocytosis of aged (apoptotic) red blood cells, by recognition of a phospho-Tyr in the HARE CD [27].

The 72-amino acid C-terminal tail of HARE (Y^2480^–L^2551^ in full-length Stab2) contains at least four endocytic motifs, an unusually high number: YSYFRI^2485^ (*M1*), FQHF^2495^ (*M2*), NPLY^2519^ (*M3*), and DPF^2534^ (*M4*). Surprisingly, three of these four motifs (*M1*,* M2, *and* M3*) mediate endocytosis of HA, with* M3* being the most active motif [26]. All three motifs participate in total coated pit targeting of HARE-HA complexes, and no single motif is required for uptake if the other functional motifs are present. The HARE CD motif network responsible for targeting the receptor to coated pits reflect either a very high level of redundancy or the presence of multiple distinct endocytic and signaling pathways. Our objective here was to identify the endocytic motifs responsible for HARE-Hep endocytosis. The results indicate that* M1*,* M3*, and* M4* are utilized for Hep endocytosis, which is a different subset of three motifs compared to that used for HA, and that in the absence of other motifs, only* M3* is able to mediate the endocytosis of both HA and Hep; HARE containing* M2* or* M4* alone did not mediate HA or Hep endocytosis.

## 2. Methods

### 2.1. Reagents, Buffers, Stable Cell Lines, and Normalization

Flp-In 293 cells, FBS, DMEM, hygromycin B, Zeocin, Lipofectamine 2000, glutamate, plasmid expression vectors, and super-competent TOP10* Escherichia coli* were from In-Vitrogen (Carlsbad, CA). Stable cells expressing HARE and HARE-mutants were generated as described previously [26, 28]. Hep was from Celsus (Cincinnati, OH) or Sigma-Aldrich (St. Louis, MO). Low endotoxin HA, made by bacterial fermentation, was from Genzyme Corp. (Cambridge, MA). Dynasore was from Sigma-Aldrich. Sodium ^125^I-iodide (100 mCi/mL; specific activity of >0.6 TBq/mg) in NaOH and PD-10 columns were from GE/Amersham Biosciences (Piscataway, NJ). Streptavidin (SA) was from Pierce (Rockford, IL). Preparation and quantification of biotinylated and iodinated ligands and the compositions of other buffers were described previously [13, 29, 30]. Other materials, reagents, and kits were obtained as described [26] or were from Sigma-Aldrich. HARE cDNA constructs and vectors for creation of stably transfected Flp-In 293 cell lines expressing wildtype (WT) HARE or HARE mutants with single or multiple endocytic motif deletions or site-specific substitutions were described previously [26, 28]. All recombinant HARE proteins contain C-terminal V5 and His_6_ epitope tags. Endocytosis Medium is DMEM with 0.05% BSA. In all experiments, the results among different HARE-expressing cell lines were normalized for HARE expression based on Western blot quantification of equal lysate protein samples [26]. Binding or endocytosis result values are expressed as the mean ± SE fmol/10^6^ cells/HARE.

### 2.2. ^125^I-SA•b-Hep Binding and Endocytosis Assays

Cells expressing WT HARE, HARE-mutants, or EV were grown in DMEM with 8% FBS and 100 *μ*g/mL hygromycin B (complete medium) in 12-well tissue culture plates for at least 2 days prior to experiments. They were processed for binding or endocytosis assays at 90–95% confluence. Radiolabeled ^125^I-SA•b-Hep or ^125^I-SA•b-HA complexes were prepared [13] using a 2 : 1 molar ratio of b-GAG : ^125^I-SA and were incubated in 0.5 mL of Endocytosis Medium for 1 h on a rotary mixer at 22°C just prior to the experiment. For nonspecific binding controls, the same amounts of ^125^I-SA and free biotin were used. ^125^I-Complexes were diluted in Endocytosis Medium to the final concentrations indicated. Cells were washed with Hanks' balanced salts solution and incubated at 37°C for 1 h with Endocytosis Medium (no serum) to allow HARE-mediated internalization of any bound serum glycosaminoglycans. The medium was aspirated and replaced with Endocytosis Medium containing 50 nM preformed complexes of ^125^I-SA with b-Hep or b-HA with or without a 50-fold excess of unlabeled ligand as competitor. The cells were then incubated either at 37°C for 1, 2, or 4 h to assess the rate of endocytosis or at 4°C for 2 h with or without 0.055% digitonin to assess total cellular or surface binding, respectively [31].

Nonspecific binding of ^125^I-SA was also assessed in parallel samples by incubating cells with ^125^I-SA•biotin complexes. The medium was removed by aspiration, and cells were washed three times (2 mL each) with cold Hanks' balanced salts solution to remove unbound ligand and solubilized in 1 mL 0.3 N NaOH. Radioactivity was measured using a Packard Cobra II gamma counter and lysate protein content was determined by the method of Bradford [32] using bovine serum albumin as standard. For each cell line, including EV, the binding of ^125^I-SA•biotin was subtracted from the binding of ^125^I-SA•b-ligand to correct for nonspecific binding of SA.

### 2.3. Treatment with Dynasore or Sucrose

WT, HARE mutants, or EV cells were preincubated in Endocytosis Medium as noted above and then incubated at 37°C for 30 min with DMSO alone or 300 *μ*M dynasore, as indicated. ^125^I-Complexes in Endocytosis Medium were then added to a final concentration of 50 nM and the cells were incubated at 37°C for 4 h. For hyperosmolar treatment, preincubated cells were further incubated in Endocytosis Medium with or without 0.45 M sucrose at 37°C for 30 min. After 30 min, medium was removed, and Endocytosis Medium with or without 0.45 M sucrose containing 50 nM ^125^I-ligand was added and the cells were incubated at 37°C for 4 h. The medium was aspirated and cells were washed three times (2 mL each) with cold Hanks' balanced salts solution to remove unbound ^125^I-ligand and processed as noted above.

### 2.4. Statistical Analysis

At least 2–4 independent experiments were performed in triplicate (*n* = 6–12) and combined data are presented as the mean ± SE. All regression lines had correlation coefficients ≥0.97 and experimental and control results were compared by unpaired Student's *t*-tests using SigmaPlot v10 (Systat Software, Inc., Point Richmond, CA). Values of *P* < 0.05 were considered statistically significant.

## 3. Results

**Figure 1:**
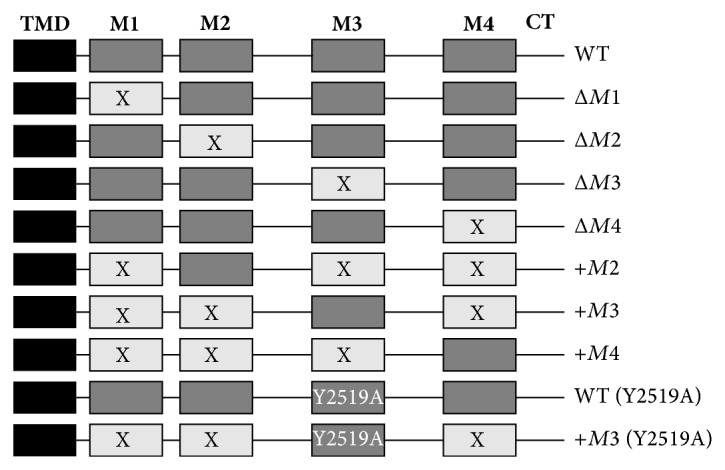
HARE CD mutants with different combinations of the four endocytic motifs. The diagram illustrates the various combinations of HARE CD motifs (*M1, M2, M3,* and* M4*) present (dark gray boxes) or deleted (light gray boxes with X) in the panel of stable HARE-CD variant cell lines used here. The single transmembrane domain (TMD, black box), C-terminal region (CT), and presence of the site-specific Y2519A mutation in* M3* are indicated.

HARE and Stab2 are scavenger receptors that bind and clear 14 different ligands, including seven glycosaminoglycans, from lymph and blood. We designate the full-length 315 kDa protein as Stab2 and HARE as the 190 kDa isoform generated by an unknown proteolytic mechanism [33]. Both HARE/Stab2 are the main systemic clearance receptors for HA and presumably Hep, in all mammals studied [34–37]. HARE is the predominant Stab2-related protein expressed in sinusoidal endothelial cells of lymph node and liver, the main systemic clearance tissues [10, 38]. Although both HA and Hep are anionic glycosaminoglycans, they bind to discrete and nonoverlapping sites in the HARE ectodomain [2]. HA binding requires the Link domain, which it likely binds to directly, whereas Hep binds to an uncharacterized site and binding is unaffected by deletion of the Link domain [2]. Since, HA and Hep bind to different sites, we wanted to determine if HARE utilizes the same subset of three redundantly functional endocytic motifs for Hep endocytosis as found previously for HA endocytosis [26]. Most of the CD mutants used here had been characterized previously for their HARE-mediated HA binding and uptake ability. Two additional single-motif containing CD mutants were created for the present study (+*M2* and +*M4*) to obtain a set of HARE CD variants expressing only one of the four motifs (e.g., Δ*M1M2M4* = +*M3*); the panel of CD mutants used is shown schematically in Figure 1. We were not successful in creating cell lines expressing only motif* M1*.

### 3.1. Cell Surface and Total Hep Binding Are Similar among Multiple HARE CD Mutants

To understand further the importance of human HARE having the ability to internalize both HA and Hep, we wanted to determine which of the four CD endocytic motifs were functional for each ligand. We previously found that HARE expression levels, as well as HA binding to surface and intracellular HARE, were similar to WT in a panel of stable Flp-In 293 cell lines expressing various CD-mutants [26]. Here we used a set of variant cell lines, expressing HARE mutants that were either single-motif deletions or containing a single-motif (i.e., three motifs deleted). To determine whether the cellular HARE distribution of Hep binding was affected in any of the variants, we compared ^125^I-SA•b-Hep binding at 4°C to cell surface or total cellular HARE (cell surface and intracellular receptors) in the various HARE CD-mutant cells. Total and surface binding were monitored in the presence or absence of digitonin, respectively, under conditions that selectively permeabilize endocytic, but not nuclear, mitochondrial or lysosomal compartments [31, 39]. Since Hep nonspecifically binds to many cell surface and intracellular proteins, the binding of Hep by EV cells is higher relative to WT cells than the nonspecific binding of HA [13, 26]. Only small amounts of ^125^I-SA•biotin (e.g., <1% of ^125^I-SA•b-Hep values) bound to cells and this did not increase with time [13]. In contrast, ^125^I-SA•b-Hep uptake was time-dependent and linear over 4 h, as in Figure 3.

**Figure 2:**
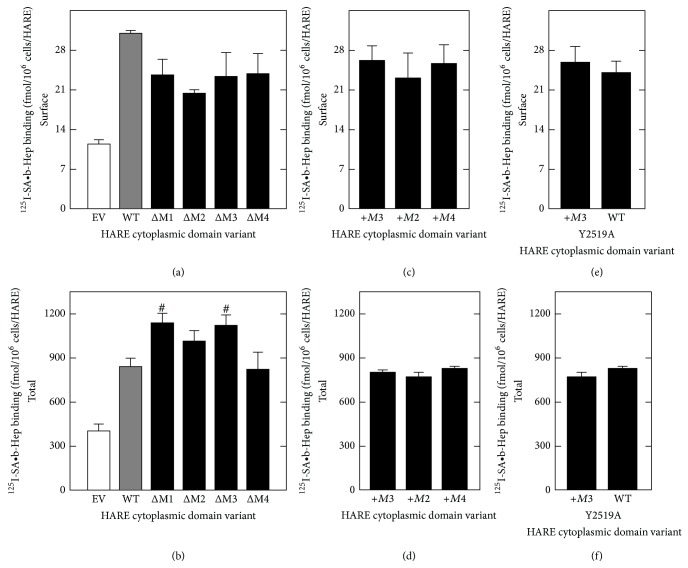
Hep binding to cell surface and total HARE in CD variants. Cells expressing human HARE (WT), the indicated HARE CD mutants, or EV were grown, washed, and preincubated in Endocytosis Medium at 37°C for 1 h to allow clearance of serum-derived glycosaminoglycans bound to HARE. Cells were chilled to 4°C, washed, and incubated with ^125^I-SA•b-Hep complexes at 4°C and processed as described in Methods section to determine cell surface (a, c, e) or total cellular (b, d, f) specific ^125^I-SA•b-Hep binding. Values are means ± SE (*n* = 6–9) and significant differences (assessed by Student's *t*-test) between WT and a HARE CD variant are indicated: ^#^ *P* < 0.05.

Cell surface (Figures 2(a), 2(c), and 2(e)) and total (Figures 2(b), 2(d), and 2(f)) ^125^I-SA•b-Hep binding to WT or HARE CD-mutant cells were 2-3 times greater than to EV cells.As expected, the distribution of ^125^I-SA•b-Hep binding sites between surface and internal was similar to that for HA binding in WT and the HARE CD mutants [26]. HARE is a constitutively active receptor involved in continuous and repeated cycles of ligand internalization and the HARE recycling time of 7–9 min [28, 40] is similar to that of other constitutively active recycling receptors [13, 41, 42]. The majority of recycling receptors, including HARE [28, 33], are localized in intracellular endocytic and recycling compartments. Thus, Hep total binding (surface and internal) by WT or CD-mutant cells was much greater than surface binding, as expected. Among the group of nine CD-mutants examined, there were no significant differences in Hep surface binding (Figure 2 top panels), confirming that deletion of one or more endocytic motifs did not alter the dynamic ongoing movement of HARE to and from the cell surface; the steady-state surface receptor pool was similar among a set of HARE variants. Total Hep binding was identical to WT among the set of nine HARE mutants except for Δ*M1* and Δ*M3* (Figure 2(b)), which were significantly higher (*P* < 0.05).

### 3.2. Internalization of ^125^I-SA•b-Hep by HARE Single-Motif Deletion Mutants

**Figure 3:**
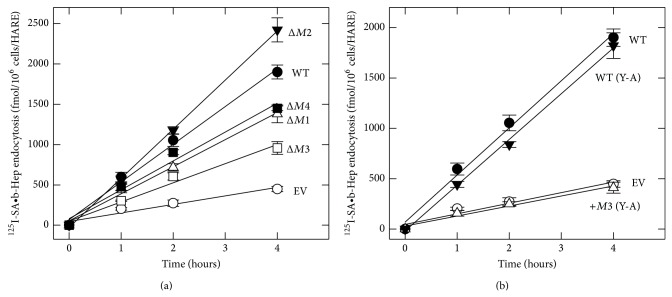
Hep endocytosis by cells expressing WT or HARE CD variants. Cells expressing WT (●), EV (○), or HARE CD mutants ((a) Δ*M1* (Δ), Δ*M2* (▼), Δ*M3* (□), and Δ*M4* (■) and (b) +*M3* (Y2519A) (Δ), and WT (Y2519A) (▼)) were pretreated and incubated with ^125^I-SA•b-Hep complexes at 37°C as in Figure 2 for the indicated times to assess uptake rates as described in Methods section. Values are means ± SE (*n* = 9) and all linear regression lines had correlation coefficients ≥0.97.

To assess the contributions of the various endocytic motifs to the kinetics of Hep endocytosis, cells expressing WT, HARE-mutants, or EV were incubated at 37°C with ^125^I-SA•b-Hep for different times (Figure 3). Partial impairment of ^125^I-SA•b-Hep endocytosis relative to WT cells occurred in Δ*M1* or Δ*M4* cells; HARE-specific uptake (WT uptake minus EV uptake) was 65% and 68% of WT rates, respectively, for Δ*M1* or Δ*M4* cells (Figure 3(a)). Cells expressing the Δ*M3* mutant showed even greater impairment of HARE-specific Hep endocytosis (35% of WT), indicating that* M3* is responsible for more targeting to coated pits than* M1* or* M4*. Surprisingly, Δ*M2* cells did not show a defect in HARE-specific Hep endocytosis, but rather a 35% increase in HARE-specific uptake compared to WT as though* M2* itself had an inhibitory effect on Hep uptake. Thus as found for HA endocytosis, three of the four motifs are involved in Hep uptake and no particular motif is absolutely required for Hep endocytosis, if the other three motifs are present. However, the subset of active motifs for Hep uptake (*M1*,* M2,* and* M3*) was not the same as that for HARE-HA complexes (*M1*,* M3,* and* M4*). Although* M1* and* M3* are used similarly for both HA uptake and Hep uptake, a different third motif is utilized by HARE for Hep (*M4*) versus HA (*M2*) endocytosis.

### 3.3. The Role of Y^2519^ in HARE-Mediated Internalization of ^125^I-SA•b-Hep

Since it is well known that phosphorylated Tyr residues in NPXY motifs are important in signaling pathways [43, 44], we wanted to identify further the importance of Y^2519^ in NPLY^2519^ for targeting HARE-ligand complexes to coated pits. We used two CD-mutant HARE cell lines, one with only a Y-to-A substitution, WT (Y2519A), and the other with the same substitution in the Δ*M1M2M4* background, +*M3* (Y2519A). WT (Y2519A) cells showed no significant defect in ^125^I-SA•b-Hep endocytosis (95% of specific WT uptake), whereas +*M3* (Y2519A) cells were identical to EV cells, showing complete impairment of Hep endocytosis (Figure 3(b)). The results show that Tyr in the HARE NPLY motif is critical for targeting to coated pits by +*M3* cells, but it is not required if HARE has functional* M2* and* M4* motifs; these motifs compensate for a potential defect in* M3*. The data are consistent with either the ability of NPLA^2519^ to retain targeting function in the presence of, but not the absence of, the two other Hep•HARE targeting motifs or the ability of* M1* and* M4* to compensate for NPLA^2519^ and perform the targeting function.

### 3.4. Internalization of Hep and HA by HARE Single-Motif Containing Mutants

**Figure 4:**
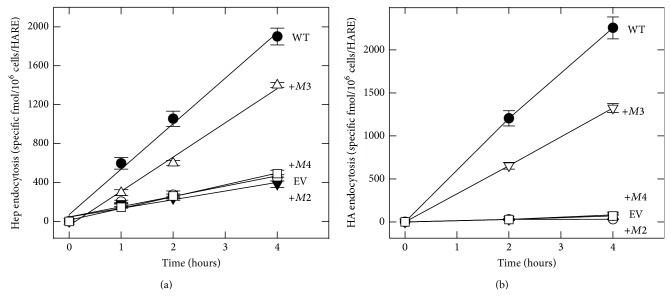
HARE mediates the endocytosis of HA or Hep in the presence of* M3* alone, but not in the presence of* M2* or* M4* alone. Cells expressing WT HARE (●), EV (○), or single-motif containing HARE CD variants +*M2* (▼), +*M3* (Δ in (a); *▽* in (b)), or* M4* (□) were grown and treated as in Figure 2, incubated at 37°C for the indicated times with ^125^I-labeled Hep (a) or HA (b), and processed to quantify uptake as described in Methods section. Values are means ± SE (*n* = 9) and all linear regression lines had correlation coefficients ≥0.97. The three symbols for EV, +*M2,* and +*M4* cells overlap at essentially identical positions in the bottom lines of each panel.

To address how multiple motifs function together to facilitate Hep endocytosis, we examined ^125^I-SA•ligand uptake in cells expressing different triple-motif deletions so that only single motifs remained (Figure 4). Interestingly, ^125^I-SA•b-Hep endocytosis by +*M2* or +*M4* cells was severely impaired by ≥95%. In contrast +*M3* cells retained 65% of the HARE-specific endocytic capability of WT cells, an effect similar to the single-motif deletions Δ*M1* or Δ*M4*. Based on studies with the single-motif deletion variants, especially Δ*M3* cells, we expected that all three HARE CD variants containing only* M2, M3,* or* M4* would be able to target HARE-Hep complexes to coated pits and mediate effective uptake. Since* M2* does not participate in Hep uptake (Figure 3(a)), we expected +*M2* cells to be defective in Hep endocytosis. However, the inability of +*M4* cells to take up Hep was unexpected, since this motif is functional in WT cells. The unexpected functional differences among the single-motif containing HARE variants are not ligand specific, as the same pattern was observed when HA endocytosis was examined (Figure 4(b)). Again, +*M2* cells (expected to be active; Figure 4(a)) or +*M4* cells (expected to be inactive) were identical to EV cells; they were both unable to internalize HA, indicating the lack of coated pit targeting and uptake. In contrast +*M3* cells showed ~60% of the endocytic capability of WT cells, a very similar result to that for Hep uptake (Figure 4(a)). Thus, although both HA (data not shown) and Hep (Figures 2(c) and 2(d)) bind equally well to HARE variants with only a single M2, M3 or M4 motif and these variants show similar surface-internal distributions (Figure 2), only* M3* by itself is able to target HARE-ligand complexes to coated pits and mediate efficient uptake. The quantitative and relative rates of ^125^I-SA•b-Hep endocytosis of the various HARE CD mutants are summarized and compared to the values for HA uptake [26] in Table 1.

### 3.5. Internalization of Hep or HA by HARE CD Mutants Is Inhibited by Hyperosmolarity

**Figure 5:**
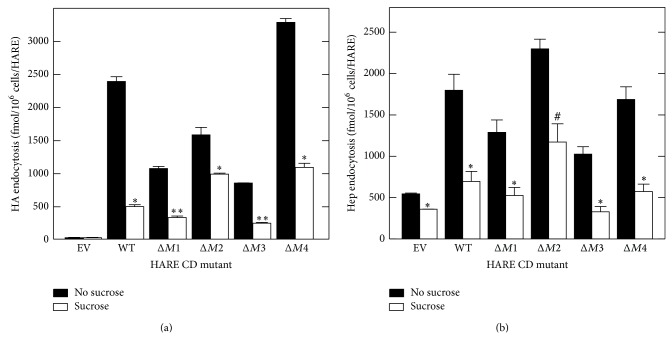
Endocytosis of HA and Hep by HARE CD variants is blocked by hyperosmolar conditions. Cells expressing EV, WT, or the indicated single-motif deletion HARE CD mutants were grown and pretreated as in Figure 2 and then preincubated at 37°C for 30 min with Endocytosis Medium with (white) or without (black) 0.45 M sucrose. The cells were then incubated with ^125^I-labelled HA (a) or Hep (b) at 37°C for 4 h and processed as described in Methods section. Values are means ± SE (*n* = 6) and significant differences (assessed by Student's *t*-test) between control and sucrose-treated samples are indicated: ^#^ *P* < 0.05; ^*^ *P* < 0.005; ^**^ *P* < 0.0005.

The unexpected behavior of HARE single-motif containing variants prompted us to verify that the various HARE CD variants mediate endocytosis using a clathrin coated pit pathway, as shown previously for native and recombinant WT HARE [28, 40, 45]. Hyperosmolar conditions inhibit clathrin assembly into coated pits and, thus, clathrin-dependent internalization of many plasma membrane receptors [46–48]. To verify further that ligand uptake by the various HARE CD-mutant cells is clathrin-dependent, we assessed the effects of hyperosmolarity on endocytosis using medium containing 0.45 M sucrose. Hyperosmolar sucrose treatment blocked internalization of HA by ~77% in WT cells compared to control (untreated) cells and by 40–70% in the single-motif deletion HARE CD mutants (Figure 5(a)). Similar results were obtained for the effects of hyperosmolarity on Hep uptake (Figure 5(b)). Overall, the results confirm that Hep and HA internalization by the various HARE CD mutants occurs via clathrin-coated pit pathways.

### 3.6. Inhibition of Dynamin Activity Blocks HA or Hep Endocytosis by HARE

**Figure 6:**
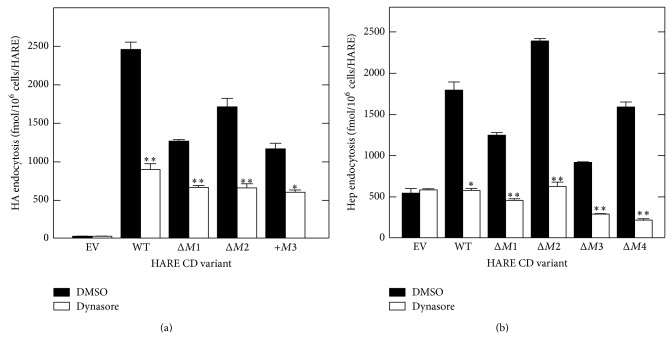
Endocytosis of HA and Hep by HARE CD variants is blocked by a dynamin inhibitor. WT cells were washed and preincubated in Endocytosis Medium as in Figure 2 and pretreated in medium with DMSO alone (black) or with 300 *μ*M dynasore (white) at 37°C for 30 min. The medium was then replaced with fresh media containing DMSO alone or dynasore and ^125^I-labelled HA (a) or Hep (b). The cells were incubated at 37°C for 4 h and specific cell-associated ligand was determined as noted in Methods section. Values are the means ± SE (*n* = 3) and significant differences (Student's *t*-test) between treated and control samples are indicated: ^*^ *P* < 0.005; ^**^ *P* < 0.0005.

Many endocytic pathways in mammalian cells, including those involving clathrin-coated pits, phagocytosis, and caveolae, require the molecular motor protein dynamin for vesicle formation [49]. Dynasore is a small cell-permeable chemical that specifically inhibits the GTPase activity of dynamin and interferes with dynamin-dependent endocytic pathways [50]. Although not absolutely specific for clathrin-mediated uptake, dynasore should inhibit HARE-mediated uptake that occurs via coated pits. Dynasore treatment significantly inhibited HA uptake by HARE CD variants with alterations in the motif subset involved in HA uptake, compared to DMSO-alone controls (Figure 6(a)). Similarly, dynasore inhibited Hep endocytosis by ~70–85% in several HARE CD mutants of the motif subset involved in Hep uptake (Figure 6(b)). As expected, the above dynasore and hyperosmolar sensitivity results indicate that the various HARE CD mutants mediate HARE-Hep and HARE-HA endocytosis by dynamin-dependent clathrin-coated pit pathways.

## 4. Discussion

Full-length Stab2 and 190 kDa HARE (the C-terminal half of Stab2) are the primary scavenger receptors for systemic clearance of multiple structurally distinct ligands (most of which are derived from tissue biomatrix degradation or cell debris) including HA, Hep, chondroitin sulfates (types A, C, D, and E), dermatan sulfate, advanced glycation end products, acetylated or oxidized LDL, collagen N-terminal propeptides, and *α*M*β*2 and *α*5*β*5 integrins [2, 4, 13, 28, 45, 51–54]. In addition, apoptotic cells and debris are rapidly cleared from blood and lymph by macrophages and sinusoidal endothelial cells via HARE-mediated binding to phosphatidylserine and then phagocytosis [3, 55]. Both functional receptor isoforms are expressed in sinusoidal endothelial cells of liver, lymph node, spleen, and bone marrow with the 190 kDa HARE being the predominant species [10]. Both are also expressed in some specialized tissues, such as corneal and lens epithelium, heart valve mesenchymal cells, epithelial cells in renal papillae, and oviduct [5]. HARE-mediated endocytosis of HA [56] or Hep [57] activates intracellular signaling leading to activation of ERK1/2 and NF-*κ*B stimulation of gene expression. Uptake of the HARE ligands dermatan sulfate and acetylated LDL [57] and phosphatidylserine [55] also activated NF-*κ*B mediated gene expression, whereas chondroitin sulfates types A, C, D, and E did not. Although all 9 of these ligands are effectively endocytosed, ERK1/2 and NF-*κ*B signaling pathways are activated by only about half of them.

We proposed that Stab2 and HARE have two important physiological functions: (i) to clear and degrade multiple ligands reflecting the status of tissue biomatrices, as first reported for HA, and (ii) to serve as a* Tissue Stress Sensor* System [58] that responds to the amounts and ratios of multiple biomatrix ligands via a signal transduction network that leads to the secretion of TGF-*β* [59] and other, yet to be identified, factors such as pro- or anti-inflammatory cytokines. The physiological importance of HARE/Stab2 for HA homeostasis was verified in Stab2 knockout mice, which have impaired systemic clearance of HA resulting in abnormally high circulating HA levels [60]. Cytokine profiles have not yet been determined in these animals. HARE may also act as a homing receptor for human prostate tumor cells, allowing metastasis to lymph nodes [61] and likely other HARE-expressing tissues, such as liver and bone marrow. Metastasis was >95% blocked by treating mice with a specific anti-HARE HA-blocking antibody.

It is well established that HARE-HA uptake is clathrin coated pit-mediated [40, 45], and this was confirmed for Hep uptake in various HARE CD mutants based on the inhibition of ligand uptake in cells treated with either the dynamin inhibitor dynasore or sucrose, under hyperosmolar conditions (Figures 5 and 6). Many endocytic receptors utilize a single CD motif for endocytosis, such as YXX*φ* (e.g., transferrin and asialoglycoprotein receptors [62, 63]) or NPXY (e.g., LDL, insulin, and EGF receptors [64, 65]). To our knowledge few other, if any, receptors contain multiple different endocytic motifs that are cooperatively utilized for endocytosis. For example, LDL receptor-related protein contains five possible endocytic motifs (1, YXX*φ*; 2, NPXY; and 2, LL), but only YXX*φ* is utilized as the dominant endocytic signal [66]. HARE is unusual and possibly unique in having four different functional endocytic motifs and in utilizing subsets of three motifs for the uptake of HA and Hep.

**Figure 7:**
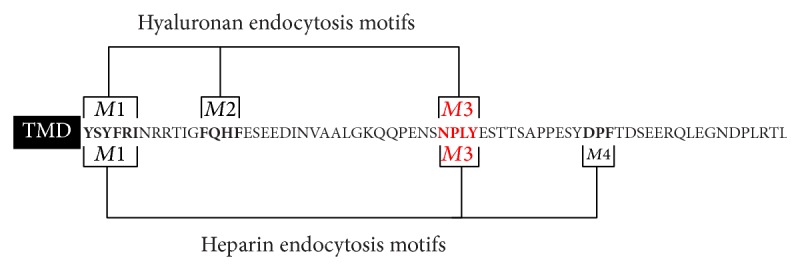
Different sets of HARE CD endocytic motifs are functional during HA versus Hep endocytosis. The four HARE endocytic motifs (boldface) examined are denoted by* M1* (YSYFRI^2485^),* M2* (FQHF^2495^),* M3* (NPLY^2519^), and* M4* (DPF^2534^). The two different subsets of three of these motifs active in the coated pit mediated endocytosis of HA (*M1, M2,* and* M3*) or Hep (*M1, M3, *and* M4*) are highlighted by brackets.* M3* is highlighted in red to indicate its special significance as the only motif of the three we were able to test (*M2, M3,* and* M4*) that enabled HARE to target coated pit mediated endocytosis of both HA and Hep.

An unexpected finding in this study was that HARE utilizes a different subset of three motifs for the endocytosis of Hep compared to HA (Figure 7). Three of the four endocytic motifs in the HARE CD (*M1* (YSYFRI^2485^),* M3* (NPLY^2519^), and* M4* (DPF^2534^)) are utilized for Hep internalization. In contrast, a different subset of three motifs (*M1*,* M2* (FQHF^2595^), and* M3*) is utilized for HA endocytosis [26]. This result and the previous finding that Hep and HA bind to independent nonoverlapping sites in the HARE ectodomain [2] indicate that the binding of HA or Hep may create distinct conformational states within the intracellular CD that promoted differential recognition of endocytic motifs* M2* and* M4* by the relevant adaptor proteins. Different conformational or multimeric states of the intracellular CD could favor efficient binding of particular adaptor proteins to specific motifs. The CD conformation of HARE-HA complexes may allow* M2* recognition by an appropriate adaptor protein, but not* M4* recognition, whereas the CD conformation of HARE-Hep complexes may allow* M4* recognition by an appropriate adaptor protein, but not* M2* recognition. Consistent with the idea that binding in the ectodomain may influence intracellular signaling, Hep does not bind within the HA-binding HARE Link domain, whereas both HA and Hep bind to the Link domain of TSG6 [67].

The consequences of this differential mechanism of Hep versus HA endocytosis are unknown but might include different downstream signaling events or trafficking outcomes for a portion of the internalized pool of Hep or HA. The impairment of HA or Hep endocytosis due to a single-motif deletion was not compensated by the other two functional motifs, indicating that each motif mediates targeting and endocytosis by a distinct independent and saturable pathway, perhaps through a subset of coated pits. If true, this has significant implications for possible independent concurrent signaling pathways mediated by different HARE-ligand complexes. One difference between the signaling stimulated by HA uptake and the signaling stimulated by Hep uptake is that HA signaling is very size-dependent. Only HA sizes between 40 kDa and 400 kDa are able to activate HARE-mediated ERK1/2 and NF-*κ*B signaling pathways; smaller or larger HA is endocytosed but does not activate signaling [11]. HARE-Hep activation of both signaling pathways is independent of Hep size [57]. Perhaps the use of different motif subsets for HA and Hep uptake is related to the mechanism by which HA size dependence is achieved during internalization of HARE-HA complexes.

The results indicate that each subset of three motifs participates in the total uptake of HA or Hep, but that the nature of their cooperation is unequal and complicated. Although the loss of only* M3* (in Δ*M3* cells) impaired Hep or HA endocytosis by ~40%, indicating that* M3* shares one-third of the Hep uptake burden, the loss of the other two Hep uptake motifs* M1* and* M4* (in +*M3* cells) only decreased endocytosis by the same amount, 35%. This was a surprising functional difference among the three motifs, since they appear to function together when all are present, but only one can function if alone. Hep and HA endocytosis were completely eliminated in +*M3* (Y2519A) cells, showing that Tyr^2519^ is important for the endocytic process mediated by* M3* alone. In WT (Y2519A) cells there was essentially no effect on uptake of either ligand. However, ongoing studies show that WT (Y2519A) cells are completely unable to activate NF-*κ*B during uptake of HA, Hep, dermatan sulfate, or acetylated LDL [57]. Thus, Tyr^2519^ is critical for signaling to downstream effectors, when the receptor is endocytosing loaded cargo, but it is not needed for just cargo endocytosis alone.

Further studies are required to define the adaptor proteins (e.g., Gulp or AP-2) that interact with the four endocytic motifs in the HARE CD and to understand the biological relevance of the complex coated pit targeting network and how it is coupled to signal transduction for a subset of internalized ligands.

## Figures and Tables

**Table 1:** Endocytosis of Hep or HA by WT or HARE CD mutants.

HARE variant	Hep endocytosis (fmol/10^6^ cells/HARE/h)	Specific Hep endocytosis (%)	Specific HA endocytosis (%)	HA endocytosis (fmol/10^6^ cells/HARE/h)
EV	110 ± 10	0	0	29.1 ± 2.8
HARE (WT)	480 ± 20	100	100	1204 ± 89
Δ*M1 *	350 ± 30^*^	65	51	—
**ΔM2 **	610 ± 40^*^	**135**	**61**	—
Δ*M3 *	240 ± 20^**^	35	44	—
**ΔM4 **	360 ± 10^*^	**68**	**119**	—
+*M2 *	111 ± 30	0	0	29.7 ± 1.2^**^
+*M3 *	350 ± 10^*^	65	58	—
+*M4 *	129 ± 10	5	0	28.2 ± 12.2^**^
+*M3* (Y2519A)	100 ± 10^**^	0	5	—
WT (Y2519A)	460 ± 30	95	94	—

EV, WT HARE, or the indicated HARE CD mutant cells were assessed for their ability to endocytose ^125^I-labeled Hep or HA specifically and results were normalized to total protein (cell number) and HARE expression level relative to WT as described in Methods section. Values are the mean ± SE (*n* = 6–12) rate of endocytosis or the rate of specific endocytosis (uptake by WT cells minus uptake by EV cells) relative to WT as 100%; significant differences compared to WT are indicated: ^*^ *P* < 0.005; ^**^ *P* < 0.0005. The relative specific HA endocytosis values for the CD variants examined previously [26] are included (third column), along with the HA values for +*M2* and +*M4* cells determined here (far right column), for comparison to the Hep endocytosis values. The single-motif deletion mutant cells (Δ*M2 *and Δ*M4*) that show differential involvement in HA versus Hep endocytosis are highlighted (boldface font).

## Abbreviations

b-: A biotinyl group
CD: Cytoplasmic domain
EV: Empty vector
HA: Hyaluronic acid, hyaluronate, and hyaluronan
HARE: 190 kDa human hyaluronic acid receptor for endocytosis
Hep: Heparin
*M1*: HARE CD motif 1 (YSYFRI)
*M2*: HARE CD motif 2 (FQHF)
*M3*: HARE CD motif 3 (NPLY)
*M4*: HARE CD motif 4 (DPF)
SA: Streptavidin
Stab2: Stabilin-2.
